# Is It Time to Redefine Fetal Decelerations in Cardiotocography?

**DOI:** 10.3390/jpm12101552

**Published:** 2022-09-21

**Authors:** Serena Xodo, Ambrogio P. Londero

**Affiliations:** 1 Department of Gynecology and Obstetrics, School of Medicine of Udine, 33100 Udine, Italy; 2 Academic Unit of Obstetrics and Gynaecology, Department of Neuroscience, Rehabilitation, Ophthalmology, Genetics, Maternal and Infant Health, University of Genoa, 16132 Genova, Italy

**Keywords:** fetal physiology, FHR decelerations, bradycardia, labor, cardiotocography, peripheral chemoreflex, myocardial hypoxia

## Abstract

Historically, fetal heart rate (FHR) decelerations were classified into “early”, “late”, and “variable” based on their relationship with uterine contractions. So far, three different putative etiologies were taken for granted. Recently, this belief, passed down through generations of birth attendants, has been questioned by physiologists. This narrative review aimed to assess the evidence on pathophysiology behind intrapartum FHR decelerations. This narrative review is based on information sourced from online peer-reviewed articles databases and recommendations from the major scientific societies in the field of obstetrics. Searches were performed in MEDLINE/PubMed, EMBASE, and Scopus and selection criteria included studies in animals and humans, where the physiology behind FHR decelerations was explored. The greater affinity for oxygen of fetal hemoglobin than the maternal, the unicity of fetal circulation, and the high anaerobic reserve of the myocardium, ensure adequate oxygenation to the fetus, under basal conditions. During acute hypoxic stress the efficiency of these mechanisms are increased because of the peripheral chemoreflex. This reflex, activated at each uterine contraction, is characterized by the simultaneous activation of two neural arms: the parasympathetic arm, which reduces the myocardial consumption of oxygen by decreasing the FHR and the sympathetic component, which promotes an intense peripheric vasoconstriction, thus centralizing the fetal blood volume. This review summarizes the evidence supporting the hypoxic origin of FHR decelerations, therefore archiving the historical belief that FHR decelerations have different etiologies, according to their shape and relationship with uterine contractions. The present review suggests that it is time to welcome the new scientific evidence and to update the CTG classification systems.

## 1. Introduction

The goal of cardiotocography (CTG) in labor is to prevent devastating outcomes, such as perinatal death and hypoxic brain injury, which is a major contributor to disability later in life. With this belief, intrapartum CTG has been introduced into routine clinical practice. However, concerns have been recently raised about its effectiveness and safety. In 2017, a Cochrane systematic review [1] clearly demonstrated that continuous CTG during labor is associated with a decreased incidence in neonatal seizures, without impacting on cerebral palsy and perinatal death, but inevitably leading to an increased incidence in operative birth. The major international societies [2,3,4] attempted to overcome these limitations by developing a standard terminology for visual interpretation of the fetal heart rate (FHR) patterns, which is based on the analysis of four features: baseline rate, accelerations, variability, and decelerations. The decelerations are the most contentious aspect (Table 1). Based on the pioneering studies of Hon and Caldeyro-Barcia, decelerations were classified into “early”, “late”, and “variable”, according to their time-relationship with uterine contractions [5,6] The temporal relationship with contractions was supposed to be the key factor in differentiating between benign and pathological decelerations. Early decelerations were thought to result from head compression. Variable decelerations were supposed to be due to umbilical cord compression. Meanwhile, late decelerations were acknowledged to result from hypoxia. Recently, this etiological classification has been questioned by physiologists, leading to a reconceptualization of intrapartum decelerations [7,8,9,10]. The objective of this narrative review was to compare the old evidence with the new on pathophysiology behind fetal decelerations during labor.

## 2. Materials and Methods

This narrative review is based on information sourced from online peer-reviewed articles databases and recommendations from the major scientific societies in the field of obstetrics. Searches were performed in MEDLINE/PubMed, EMBASE, and Scopus from the inception of each database until August 2022. Only the articles in English were included. The selection criteria included studies in animals and humans, where the physiology behind FHR decelerations was explored. Additionally, letters to the editor or reviews where new evidence was explained were included.

## 3. Synthesis

### 3.1. Early Decelerations

Early decelerations are historically thought to result from head compression, which would occur when the fetal head starts the engagement [11]. Therefore, these decelerations are considered benign, being caused by a non-hypoxic reflex (Table 2). In 1959, Chung and Hon tried to reproduce early decelerations by mimicking the head compression effect experimentally [12]. They repetitively applied pressure to the fetal skull either with the finger or with a pessary in six patients with a cervical dilatation of at least 8 cm and no previous decelerations on CTG. They found a variable response of the FHR to vaginal examinations and to the application of pessary (a drop of the FHR below 100 bpm was observed in 12 trials only). The FHR drop was supposed to be caused by a neural reflex triggered by the augmented intracranial pressure during labor. In 1964, Paul et al. performed six head compressions on four full-term lambs and observed FHR decreases that were consistent with the reduction in the blood flow of the carotid arteries [13]. According to the authors, the vagal activation was highly likely to be due to hypoxia of the central nervous system. In 1989, Harris et al. observed no fetal decelerations when intracranial pressure was artificially increased in fetal sheep [14]. The authors showed that the fetus could maintain its cerebral perfusion despite the elevation of intracranial pressure obtained with the infusion of artificial cerebrospinal fluid. Moreover, they observed that the fall in FHR exclusively occurred when intracranial pressure increased above the mean arterial blood pressure. This finding was replicated in other experiments, shedding light on the existence of a fetal intracranial baroreflex, which provides a graded defense against rises in intracranial pressure. Unquestionably, only head compression that is severe enough to determine profound cerebral hypoperfusion is likely to trigger an FHR deceleration (Table 2). In 1992, Harris et al. tried to reproduce the fetal environment during labor, studying the fetal response to transient intracranial pressure increases. Intracranial pressure was raised above the mean arterial pressure over 45 s and then was decreased back to the baseline over 45 s. Recovery periods of 3.5 min separated the intracranial pressure variations. Animal fetuses responded to the stimuli with much smaller cardiovascular responses, suggesting that the intracranial baroreflex is attenuated when briefly and abruptly stimulated [15]. The authors further hypothesized that these defenses might involve humoral factors [15]. However, fetal responses were augmented with repeated stimulation, which is likely due to the progressive accumulation of humoral factors [10]. Overall, these experiments demonstrated that periods of head compression such as during a typical, spontaneous delivery did not critically impair cerebral perfusion and were therefore, not associated with FHR decelerations. If decelerations occurred, the autonomic cardiovascular response would be stimulated by severe cerebral hypoperfusion due to extreme increases in intracranial pressure. These decelerations are indeed not benign.

### 3.2. Variable Decelerations

Variable decelerations are thought to be the result of cord compression [11]. The unpredictability of cord compression, which depends on the position of the fetus in utero, would give rise to a deceleration that has a variable relationship with the contraction and that could be eliminated by changing the mother’s position (Table 2). Barcroft, in 1947, reproduced variable decelerations by occluding the umbilical cord of a fetal goat [16]. Thereafter, different researchers repeated the same type of experiment. In 1983, Itskowitz et al. performed a prolonged (lasting 40 s) cord compression in nine fetal lambs in utero. They measured the mean aortic pressure, the heart rate, and the umbilical blood flow through different intervals and catheters placed in the fetuses [17]. Interestingly, the authors observed FHR responses resembling variable decelerations when the umbilical blood flow was reduced by at least 50%, while bradycardia was observed with complete cord occlusion. They argued that the vagal-mediated FHR decrease was triggered by chemoreceptors’ activation in partial cord occlusion and by the activation of chemo- and baroreceptors in case of complete cord occlusion. The baroreflex was advocated as the one responsible for eliciting fetal bradycardia, as hypertension was reported to be associated with the complete interruption of umbilical blood flow and consequent hypoxemia. However, previous work by Lee and Hon in 11 human fetuses undergoing a cesarean delivery had already shown that decelerations occurring after partial and complete umbilical cord occlusion were associated with either increased, decreased, or no change in blood pressure [18]. Thus, the baroreflex mediated mechanism did not exhaustively and convincingly explain the FHR slowdown. Some authors hypothesized that the activation of the Bezold-Jarish reflex triggers variable decelerations [19,20]. When umbilical cord compression occurs, the hypovolemia resulting from umbilical vein compression leads to a decreased venous return to the fetal heart, which in turn leads to the activation of the vagal reflex giving rise to fetal deceleration. However, some considerations persuaded researchers to reject this hypothesis: first, in fetal sheep, this reflex was not activated by reduced cardiac pressures; and second, when activated, this reflex induced a delayed deceleration, which is not consistent with the typical pattern of the variable deceleration (contemporaneous to contraction) [21,22]. Recent advances in the field of fetal physiology have shown that variable decelerations originated from hypoxemia, which was caused by at least a 50% reduction in intervillous perfusion and was mediated by the peripheral chemoreflex [17,19,23] (Table 2).

### 3.3. Late Decelerations

Late decelerations are deemed the only FHR decreases resulting from uteroplacental insufficiency [24] (Table 2). Past animal and human experiments have shown that vagotomy momentarily stopped the initial phase of fetal deceleration caused by hypoxia, but that the FHR drop reappeared if hypoxia persisted [5]. These observations convinced researchers to believe that the critical decrease in the blood flow of the intervillous space gave rise to decelerations, for which the nadir occurred after the peak of the contraction and the return to the baseline heart rate resulted with a lag time of 10–20 s after the contraction had finished [11] (Table 2). Late decelerations were explained as resulting from anaerobic metabolism due to impaired gaseous exchange causing hypoxemia, hypercapnia, and acidosis [25]. Late decelerations originally described by Hon occurred before labor and were accompanied by baseline tachycardia, low variability, and absent accelerations. These decelerations are pathognomonic of poor uteroplacental perfusion, where a growth-restricted fetus is unable to adapt to small decreases in gas exchange associated with mild uterine contractures [24]. In this clinical scenario, the fetus would not adapt to labor stress. By contrast, late intrapartum decelerations are simply variable decelerations that are late in timing [10]. However, no experimental evidence supports the conclusion that these decelerations are associated with a greater physiological challenge than early or variable decelerations [26,27]. It could be hypothesized that the lag time between the contraction and the deceleration might correspond to the time necessary to impair fetal oxygenation sufficiently to trigger deceleration and then to recover.

### 3.4. New Insights into the Pathophysiology of Fetal Intrapartum Decelerations

**Figure 1:**
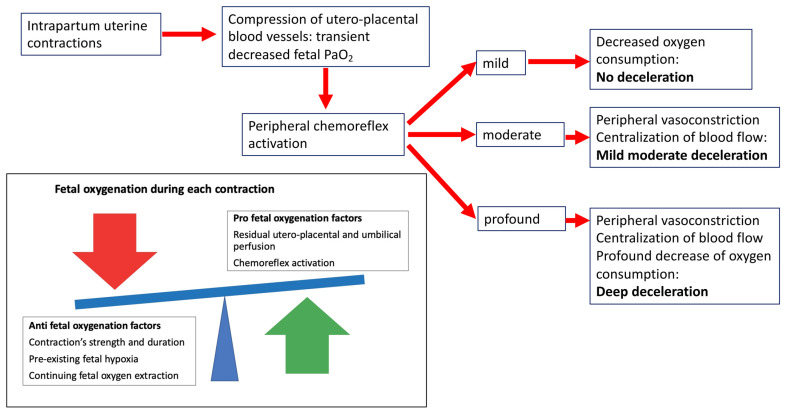
Pathophysiology of fetal decelerations. Each contraction decreases the blood flow of the intervillous space, thus leading to transient fetal hypoxemia. The peripheral chemoreflex, which is characterized by two neural arms, is activated at each contraction. The parasympathetic arm reduces the myocardial consumption of oxygen by decreasing the FHR. The sympathetic component promotes an intense peripheric vasoconstriction, thus centralizing the fetal blood volume. The combined effect of these two actions increases the fetal systemic pressure, guaranteeing the blood supply to central organs such as the heart, the brain, and the adrenal glands. The greater the hypoxic insult, the more intense is the activation of the peripheral chemoreflex. When the hypoxic insult is prolonged the deceleration is maintained by the myocardial hypoxia.

Given the rarity of birth asphyxia, the idea, erroneously established over the last 50 years, is that only some intrapartum decelerations are caused by hypoxia, while the majority does not have a hypoxic origin. Recent advances in the knowledge of fetal physiology have demonstrated that birth asphyxia is rare because the fetal tolerance to hypoxia is extraordinarily high [7,8,19]. The phrase “mount Everest in utero” perfectly depicts the ability of the fetus to survive in a hypoxic environment [16]. Under basal conditions, the fetus uses several strategies for its oxygenation. First, its hemoglobin has a greater affinity for oxygen than the adult phenotype [28]. Second, the shunts in the fetal circulation, such as the ductus venosus, the foramen ovale, and the ductus arteriosus, ensure an adequate supply of oxygenated blood to organs that are critical for survival [29]. Last, the fetus has an extremely high anaerobic reserve in the myocardium [28]. The efficiency of these basal abilities is increased during acute hypoxic stress by the following different mechanisms: consuming even less oxygen, extracting even more oxygen from hemoglobin, and making better use of this limited supply of oxygenated blood [30]. These responses are mediated by the peripheral chemoreflex, which is characterized by the simultaneous activation of two neural arms [19]. The parasympathetic arm reduces the myocardial consumption of oxygen by decreasing the FHR. Meanwhile, the sympathetic component promotes an intense peripheric vasoconstriction, thus centralizing the fetal blood volume. The combined effect of these two actions increases the fetal systemic pressure, guaranteeing the blood supply to central organs such as the heart, the brain, and the adrenal glands (Figure 1). The peripheral vasoconstriction is further maintained by humoral factors (catecholamines, vasopressin, and neuropeptide Y) [30]. Labor is characterized by brief periods of asphyxia occurring intermittently. Each contraction decreases the blood flow of the intervillous space, thus impairing the blood gas exchange and ultimately resulting in hypoxemia, hypercapnia, and a shift towards anaerobic metabolism [7,8]. The peripheral chemoreflex is activated at each contraction (Figure 1). Its maximal activation occurs when the uteroplacental blood flow is reduced by 90% [17], thus reflecting that the greater the hypoxic insult, the more intense is the response of this neural reflex. Therefore, intrapartum decelerations are the fetal response to a decreased transplacental gas exchange (i.e., hypoxic stress). Experimental studies have demonstrated that decelerations are provoked by a >50% decrease in uteroplacental blood flow [17,22]. That is why each contraction does not always correspond to a fetal deceleration. A healthy fetus can often respond to mild reductions in oxygen supply without decelerating its heart rate. Another interesting observation points out that the activation of this life-saving neural reflex has a finite duration. The peripheral chemoreflex becomes attenuated when the hypoxic insult lasts beyond 90–120 s, at which point the deceleration is maintained by the myocardial hypoxia [26]. In healthy fetuses, the effect of the peripheral chemoreflex on FHR is replaced by the effect of myocardial hypoxic insult during deep and prolonged decelerations. This critical point reflects the transition from a reassuring state, in which the fetus is able to deal with the intermittent hypoxia, to a non-reassuring state in which the fetus is decompensating. Currently, the transition to decompensation could not be captured with cardiotocography, but is the object of interest for many researchers.

### 3.5. The State of the Art of Current Intrapartum Cardiotocography

The high interobserver disagreement in the interpretation of intrapartum cardiotocographs is well recognized. Overall, the most popular classification systems agree on the first category (type I in ACOG, normal in FIGO, reassuring in NICE) and the third category (type III in ACOG, pathological in FIGO, and abnormal in NICE), but not on the second category of CTG pattern (type II in ACOG, suspicious in FIGO, and non-reassuring in NICE) (Table 1) [31]. The second category includes a broad spectrum of CTG patterns, defined by a combination or not of specific features, such as: prolonged decelerations; repetitive, variable and late decelerations; and baseline tachycardia. These patterns describe an indeterminate situation in which the fetus intermittently deals with short or prolonged periods of hypoxia and may be at risk of asphyxia if its compensation is overwhelmed. Although a moderate uniformity in defining decelerations could be recognized among the three classification systems, visual interpretation of decelerations is severely exposed to subjectivity, thus enhancing inter-observer disagreement. Additionally, some concepts at the basis of the terminology used are completely erroneous. First, given that no difference exists in the pathophysiology of FHR decelerations, they could not be divided into “benign” and “pathological.” Second, all decelerations originate from hypoxia, which is a typical feature of labor. Thus, the shape of decelerations does not suggest a causation mechanism other than hypoxia. The revolutionary view recently illustrated by Lear et al. in different papers is that brief decelerations in labor are peripheral chemoreflex responses to transient, but repeated, hypoxia [7,8,9,10,19]. The point at which the origin of FHR deceleration switches from the peripheral chemoreflex to hypoxic myocardial injury is currently not known. Much energy has been spent capturing this transition, which anticipates fetal decompensation. However, despite the intense efforts of current and past research to improve the predictive value of cardiotocography, no helpful tools have been introduced to identify fetal compromise in labor. We believe that a step forward might be represented by removing the erroneous concepts in the pathophysiology of FHR decelerations from classification systems. The fact that all decelerations originate from hypoxia should not be alarming, because the fetus actually has impressive abilities to deal with hypoxia. Not the shape and the relationship with uterine contractions, but the duration, depth, and frequency of decelerations as well as the contractions’ frequency and duration, should be considered in the broad scenario. Two different research groups have given an important contribution in this direction over the last 10 years. In 2012, Cahill et al. retrospectively analyzed the total deceleration area of the last 30 min prior to the birth, of CTG patterns in a cohort of more than 5000 women and found that the total deceleration area was the most predictive of fetal acidemia compared with other CTG features [27]. In 2014, Ugwumadu, and in 2016, Chandraharan, proposed a physiological approach to enhance the CTG’s positive predictive value for intrapartum hypoxic-ischemic injury [11,32]. Even though the classical categorization in early, late, and variable decelerations was maintained, the novelty of this approach consisted of differentiating between gradually evolving hypoxia and subacute hypoxia by focusing on fetal behavior and contraction patterns. The first type of hypoxia was described to develop progressively over time and to be labeled by a sequence of fetal events observed in the CTG: decelerations, the disappearance of accelerations, the increase in baseline HR, and the loss of variability preceding fetal decompensation. The second type of hypoxia was described as evolving rapidly, over 30–60 min, and to occur when the fetus spends more time under contractions than under resting uterine tone. However, once again, the specific point at which the fetus transitions to myocardial hypoxia is not known.

The importance of removing the wrong concepts in the definition of FHR decelerations would have practical consequences for obstetricians. Decades of teaching that early decelerations are due to head compression and therefore, innocuous, and that variable decelerations only indicate umbilical cord compression and thus may be tolerated indefinitely, has led obstetricians to failing to perceive the alarm of some labors, leading to tragic outcomes. Interestingly, a Swedish nationwide study described the possible causes of labor asphyxia in women asking for financial compensation because of suspected medical malpractice and found that negligence in the supervision of intrapartum electronic fetal monitoring occurred in 98% of the cases and the most frequently observed FHR patterns associated with cerebral palsy included multiple late decelerations [33]. These findings prove that even late decelerations could be wrongly tolerated by obstetricians, thus suggesting that there is much confusion about types of decelerations and their significance.

## 4. Limitations to the Overview

Some limitations of this overview should be acknowledged. First of all, it should be recognized that our ability to understand fetal physiology is greatly impaired by the difficulty in reproducing such complex physiological mechanisms. Second, there are considerable ethical boundaries around performing studies on fetal physiology in humans and practical challenges in carrying out high-quality studies in animals. Third, it could be criticized that the highlighting of the hypoxic origin of all FHR decelerations would raise the alarm bell and thus increase the medical intervention rate. However, the low predictive value of cardiotocography and the disproportionate operativity increase in labor are well-recognized drawbacks, which could be antagonized by improving the knowledge on fetal physiology. Therefore, we strongly believe that it is time to abandon terms like “early”, “late”, and “variable”, and to focus our attention on frequency, depth, and duration of decelerations during labor, rather than on their timing, shape, or supposed etiology. This approach might help obstetricians to have a more physiological perspective of the intrapartum fetal well-being and could be recognized as a first step towards improving the accuracy of CTG.

## 5. Conclusions

Despite recent advances in fetal physiology, the currently most-used CTG classification systems worldwide are based on outdated terminology, especially regarding fetal decelerations. It is imperative to start to look at fetal decelerations as a fetal response to hypoxic stress, which in the case of normal labor occurs intermittently and briefly. In the case of an indeterminate CTG pattern, the severity of the hypoxic stress is arguable by the pattern of decelerations (the worse when deeper and longer) and the pattern of contractions (the most dangerous when frequently occurring and longer). Nevertheless, the CTG’s predictive value of fetal compromise in labor remains poor. In conclusion, the present review suggests that it is time to welcome the new scientific evidence and to update the CTG classification systems.

## Figures and Tables

**Table 1:** Deceleration definitions according to the recommendations of the major scientific societies in the obstetric field.

Decelerations	ACOG 2009	FIGO 2015	NICE 2014
Early	Usually symmetrical gradual decrease and return of the FHR associated with a uterine contraction. A gradual FHR decrease is defined as from the onset to the FHR nadir of 30 s or more. The decrease in FHR is calculated from the onset to the nadir of the deceleration. The nadir of the deceleration occurs at the same time as the peak of the contraction. In most cases the onset, nadir, and recovery of the deceleration are coincident with the beginning, peak, and ending of the contraction, respectively.	shallow, short-lasting, with normal variability within the deceleration and are coincident with contractions. They are believed to be caused by fetal head compression and do not indicate fetal hypoxia/acidosis.	
Late	Usually symmetrical gradual decrease and return of the FHR associated with a uterine contraction. A gradual FHR decrease is defined as from the onset to the FHR nadir of 30 s or more. The decrease in FHR is calculated from the onset to the nadir of the deceleration. The deceleration is delayed in timing, with the nadir of the deceleration occurring after the peak of the contraction. In most cases, the onset, nadir, and recovery of the deceleration occur after the beginning, peak, and ending of the contraction, respectively.	U-shaped with a gradual onset and/or a gradual return to the baseline and/or reduced variability within the deceleration. Gradual onset and return occurs when more than 30 s elapses between the beginning/end of a deceleration and its nadir. When contractions are adequately monitored, late decelerations start more than 20 seconds after the onset of a contraction, have a nadir after the acme, and a return to the baseline after the end of the contraction. These decelerations are indicative of a chemoreceptor-mediated response to fetal hypoxemia. In the presence of a tracing with no accelerations and reduced variability, the definition of late decelerations also includes those with an amplitude of 10−15 bpm.	Present for over 30 min; they do not improve with conservative measures and occurring with over 50% of contractions
Variable	Visually apparent abrupt decrease in FHR An abrupt FHR decrease is defined as from the onset of the deceleration to the beginning of the FHR nadir of less than 30 s. The decrease in FHR is calculated from the onset to the nadir of the deceleration. The decrease in FHR is 15 beats per minute or greater, lasting 15 seconds or greater, and less than 2 min in duration. When variable decelerations are associated with uterine contractions, their onset, depth, and duration commonly vary with successive uterine contractions.	V-shaped and exhibit a rapid drop (onset to nadir in less than 30 s), good variability within the deceleration, rapid recovery to the baseline, varying size, shape, and relationship to uterine contractions. They constitute the majority of decelerations during labor, and translate a baroreceptor-mediated response to increased arterial pressure, as occurs with umbilical cord compression. They are seldom associated with an important degree of fetal hypoxia/acidosis, unless they evolve to exhibit a U-shaped component, reduced variability within the deceleration, and/or their individual duration exceeds 3 min (prolonged decelerations).	Dropping from baseline by 60 beats/minute or less and for 30–90 min taking 60 seconds or less to recover. They are present for over 90 min and occurring with over 50% of contractions. OR Dropping from baseline by more than 60 beats/minute or taking over 60 seconds to recover. They are present for up to 30 min and occurring with over 50% of contractions.

**Table 2:** Old versus new physiologic perspective on fetal decelerations.

Deceleration Type	Historical Belief	Why Historical Belief Is Misleading	New Insights in Pathophysiology of Fetal Decelerations
Early decelerations	-Results from head compression during fetal head engagement in the birth canal; -Considered benign and not caused by hypoxia;	-Only head compression severe enough to determine profound cerebral hypoperfusion triggers FHR decelerations; -Periods of head compression such as during spontaneous delivery do not critically impair cerebral perfusion and are not associated with FHR decelerations;	In case of extreme increases in intracranial pressure decelerations are not benign, being the fetal response to severe cerebral hypoperfusion
Variable decelerations	-Results from cord compression, therefore, having a variable relationship with contractions; -Potentially eliminable by changing the maternal position;	-The baroreflex mediated mechanism implicates that hypertension is consistently observed with complete cord occlusion, which was not experimentally confirmed; -The Bezold-Jarish reflex implicates that hypovolemia due to umbilical cord compression leads to a decreased venous return to the fetal heart, which in turn leads to the activation of the vagal reflex, thus provoking deceleration. However, in fetal sheep this reflex was not activated by reduced cardiac pressures. Moreover, when activated, this reflex induced a delayed deceleration, which is not consistent with the typical pattern of the variable deceleration.	These decelerations originate from hypoxemia, which is caused by at least a 50% reduction in intervillous perfusion, and are mediated by the peripheral chemoreflex
Late decelerations	-Results from utero-placental insufficiency.	No experimental evidence supports the conclusion that these decelerations are associated with a greater physiological challenge than early or variable decelerations.	Rhese decelerations originate from hypoxemia, which is caused by at least a 50% reduction in intervillous perfusion, and are mediated by the peripheral chemoreflex.

## Data Availability

Not applicable.
